# Cirrhosis Is an Independent Risk Factor for Mortality in Ischemic Stroke—A Nationwide Analysis

**DOI:** 10.1155/ijh/9250819

**Published:** 2025-03-19

**Authors:** Kayode E. Ogunniyi, Habib Olatunji Alagbo, Oluwaremilekun Zeth Tolu-Akinnawo, Selimat Ibrahim, Oluwaseun Dorcas Adeleke, Arun Mahtani, Derek Fan Ugwendum, Indebir Padda, Meena Farid, Toluwalase Awoyemi

**Affiliations:** ^1^Department of Internal Medicine, Richmond University Medical Center/Mount Sinai, Staten Island, New York, USA; ^2^Department of Medicine, Faculty of Medicine, University of Coimbra, Coimbra, Portugal; ^3^Department of Internal Medicine, Meharry Medical College, Nashville, Tennessee, USA; ^4^Department of Internal Medicine, Nikzar Medical Center, Ibadan, Nigeria; ^5^Department of Internal Medicine, Sumy State University, Sumy, Ukraine; ^6^Department of Internal Medicine, Feinberg School of Medicine, Northwestern University, Chicago, Illinois, USA

**Keywords:** ischemic stroke, liver cirrhosis, mortality, predictors

## Abstract

**Background and Aims:** Ischemic stroke remains a leading cause of preventable cardiovascular mortality worldwide, with emerging evidence suggesting an association between liver cirrhosis and both stroke occurrence and severity. However, the specific impact of cirrhosis on stroke-related mortality remains incompletely understood. Elucidating this relationship is crucial for improving risk stratification and early recognition of high-risk individuals.

**Methods**: We conducted a retrospective cohort study comparing ischemic stroke patients with cirrhosis to those without, using the National Inpatient Sample database for 2021. Univariate and multivariate logistic regression analyses were performed to compare various outcomes.

**Results:** A total of 536,199 discharges for ischemic stroke were included, among which 4464 had a documented history of liver cirrhosis. Discharges with cirrhosis were predominantly male (58.2%) with a mean age of 67 years, which was 2.17 years younger than those without cirrhosis. In-hospital mortality was 7% (95% CI: 5.5%–8.99%) among discharges with cirrhosis versus 4.2% (95% CI: 4.0%–4.33%) in those without.. After adjusting for cofounders in multivariate logistic regression, it was revealed that cirrhosis is associated with 69% higher mortality risk in stroke discharges (OR = 1.69, 95% CI: 1.27–2.25, *p* < 0.001).

**Conclusions:** Our study identifies liver cirrhosis as an independent risk factor for mortality among patients hospitalized with ischemic stroke. These findings underscore the necessity of incorporating proactive management strategies for liver cirrhosis into stroke care and prevention protocols, potentially improving outcomes in this high-risk population.

**Summary**

The study provides strong evidence that liver cirrhosis is an independent and significant risk factor for increased in-hospital mortality in ischemic stroke patients, highlighting the need for specialized care in this high-risk group.

## 1. Introduction

Ischemic stroke, the predominant form of cerebrovascular accident, affects approximately 700,000 individuals and accounts for over 150,000 fatalities annually in the United States [1]. It is characterized by insufficient cerebral blood flow, resulting in neurological deficits and functional impairment [2, 3]. The etiology of ischemic stroke is multifactorial, encompassing both nonmodifiable risk factors (e.g., advanced age, ethnicity, sex, and genetic predisposition) and modifiable factors (e.g., obesity, hypertension, diabetes mellitus, tobacco use, and atrial fibrillation) [1, 4]. Timely intervention, typically involving thrombolytic therapy or endovascular thrombectomy, is crucial for mitigating adverse outcomes by restoring perfusion to the affected cerebral territory [5]. However, patient prognosis varies significantly, influenced by factors such as the extent of ischemic injury, patient age, and comorbidities, with hepatic cirrhosis emerging as a particularly concerning comorbidity that exacerbates the risk of adverse outcomes, including mortality [4]. Several studies have demonstrated that hepatic cirrhosis complicates ischemic stroke outcomes and is associated with increased mortality rates [6–8]. This elevated mortality is hypothesized to be due to activated hepatic stellate cells in cirrhotic livers, which contribute to elevated levels of circulating proinflammatory cytokines, procoagulant factors, and oxidative stress markers, all of which increase the risk of mortality in ischemic stroke patients [1].

Previous investigations into the association between hepatic cirrhosis and ischemic stroke outcomes have been limited by factors such as insufficient sample sizes, potential misclassification bias, and/or inadequate control of confounding variables and disease severity [9, 10]. Given the significant impact of hepatic cirrhosis on ischemic stroke outcomes, our study is aimed at further elucidating this association to enhance risk stratification and recognition in high-risk individuals.

## 2. Materials and Methods

### 2.1. Data Source

Data for this study were obtained from the National Inpatient Sample (NIS) database for the year 2021. The NIS, part of the Healthcare Cost Utilization Project (HCUP) supported by the Agency for Healthcare Research and Quality (AHRQ), is the largest publicly available all-payer inpatient database in the United States. It is designed to produce national and regional estimates of inpatient utilization, access, costs, quality, and outcomes.

The NIS employs a stratified probability sampling design, representing all nonfederal acute care hospitals nationwide. Hospitals are stratified based on ownership/control, bed size, teaching status, urban/rural location, and geographic region. Within each stratum, a 20% probability sample of hospitals is selected, and all discharges from these hospitals are recorded and weighted to ensure national representativeness.

The database provides comprehensive discharge information, including patient demographics, primary payer, hospital characteristics, principal diagnosis, secondary diagnoses, and procedural diagnoses. For the 2021 dataset, the NIS sampling frame included data from 49 statewide data organizations (48 states plus the District of Columbia), covering more than 97% of the US population.

### 2.2. Inclusion Criteria and Study Variables

We conducted a retrospective cohort study utilizing the 2021 NIS database, focusing on adult patients (≥ 18 years) with a principal diagnosis of ischemic stroke (cerebral infarction). The study population was stratified based on the presence or absence of liver cirrhosis as a secondary diagnosis.

Key variables analyzed included the following:

a. Demographic factors: age, gender, and race
b. Hospital characteristics: bed size (small, medium, large), type, and region of location
c. Insurance type: Medicare, Medicaid, private, and others
d. Comorbidities: Charlson comorbidity index, hypertension, diabetes, chronic kidney disease, alcohol use, chronic viral hepatitis, and heart failure

Primary and secondary outcomes are outlined in subsequent sections.

We examined baseline characteristics, outcomes, and predictors of inpatient mortality.

### 2.3. Outcomes Measured

The primary outcome was in-hospital mortality among patients principally admitted for ischemic stroke with versus without liver cirrhosis. Secondary outcomes included the following:

a. Mechanical ventilation
b. Hemorrhagic transformation
c. Hemorrhagic transformation postthrombolysis
d. Vasopressor requirement
e. Discharge disposition
f. Resource utilization associated with hospitalization, defined by the average length of hospital stay and patient charges
g. Independent predictors for mortality

### 2.4. Statistical Analysis

Statistical analyses were conducted using STATA/BE 18.0, employing methods appropriate for the complex survey design of the National Inpatient Sample. Comparisons of baseline characteristics between cirrhotic and noncirrhotic populations were performed using Student's *t*-test for continuous variables and chi-square or Fisher's exact test for categorical variables, as appropriate. To account for the survey design, we utilized the survey data analysis commands in STATA, incorporating sampling weights, stratification, and clustering.

Univariate logistic regression models were initially employed to calculate unadjusted odds ratios (ORs) for primary and secondary outcomes. Subsequently, multivariate logistic regression models were constructed to adjust for potential confounders, including demographic factors, comorbidities, and hospital characteristics. These models allowed us to estimate adjusted odds ratios (aORs) and their corresponding 95% confidence intervals.

To ensure the robustness of our findings, we conducted sensitivity analyses, including stratification by cirrhosis etiology and assessment of interaction terms. All statistical tests were two-tailed, with statistical significance set at *p* < 0.05. This comprehensive statistical approach allowed for a thorough examination of the relationship between liver cirrhosis and ischemic stroke outcomes, while minimizing potential biases and accounting for the complex nature of the NIS dataset.

## 3. Results

### 3.1. Patient Characteristics

Our study included 536,199 discharges with a diagnosis of ischemic stroke in 2021, with a mean age of 69.7 ± 0.2 years. Among these, 4464 (0.83%) had concurrent liver cirrhosis, while 531,735 (99.17%) did not.  Table 1 presents the baseline characteristics of both groups.

Discharges with concurrent ischemic stroke and liver cirrhosis were younger (mean age 67.5 ± 10.8 years vs. 69.7 ± 13.7 years) and predominantly male (58.2% vs. 51.3%) compared to those without cirrhosis. The cirrhotic group had a significantly higher mean Charlson comorbidity index (5.67 ± 2.3 vs. 3.82 ± 2.1), indicating a greater burden of comorbidities. Racial distribution was similar between groups, with Caucasians comprising 67.7% of cirrhotic discharges and 66.9% of noncirrhotic discharges. Hospital characteristics and insurance status were comparable between groups, with approximately 52.7% admitted to large hospitals and 63% covered by Medicare in both cohorts. However, comorbidity profiles differed notably. Diabetes was more prevalent in the cirrhotic group (48.7%), while hypertension was the most common comorbidity (55%) in the noncirrhotic group.

These baseline differences highlight the distinct clinical profile of ischemic stroke discharges with concurrent liver cirrhosis, characterized by younger age, male predominance, and a higher comorbidity burden. These factors may contribute to the differential outcomes observed between the two groups and underscore the importance of tailored management strategies for this high-risk population.

### 3.2. Liver Cirrhosis Independently Contributes to In-Hospital Mortality in Ischemic Stroke Discharges

In-hospital mortality rates differed significantly between discharges with ischemic stroke and concurrent liver cirrhosis compared to those without cirrhosis. Among discharges with both conditions, 7.1% resulted in in-hospital mortality, whereas 4.2% of discharges with ischemic stroke alone experienced in-hospital mortality (Table 2).

After adjusting for potential confounders, the presence of liver cirrhosis was associated with a 69% increase in the odds of in-hospital mortality (aOR 1.69, 95% CI 1.27–2.25; *p* < 0.001).

**Figure 1:**
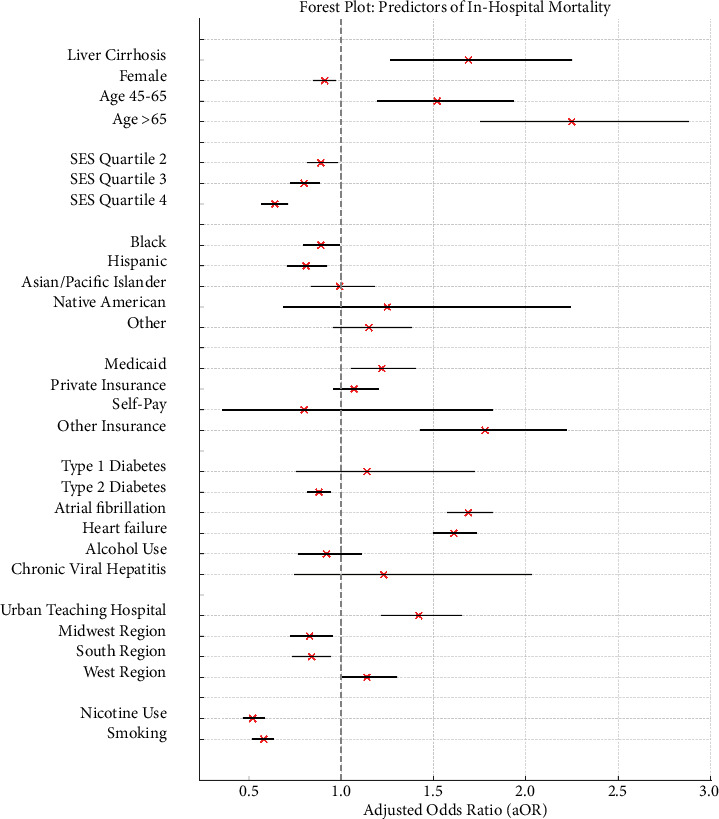
Forest plot of predictors of in-patient mortality.

Multivariate logistic regression analysis identified several independent predictors of in-hospital mortality among ischemic stroke discharges in addition to liver cirrhosis (Figure 1).

Age was a strong predictor, with increased risk for discharges for those aged 45–64 (aOR = 1.52, 95% CI 1.20–1.93, *p* = 0.001) and those over 65 (aOR = 2.25, 95% CI 1.76–2.88, *p* < 0.001). Female gender showed a slightly protective effect (aOR = 0.91, 95% CI 0.85–0.97, *p* = 0.005). Socioeconomic factors also influenced mortality risk. Discharges in higher income quartiles had lower mortality odds compared to the lowest quartile. Hospital type and location were significant predictors of mortality risk. Patients treated in urban teaching hospitals had a higher mortality risk (aOR = 1.42, 95% CI 1.22–1.65, *p* < 0.001) compared to those treated in rural or urban nonteaching hospitals. No significant difference in mortality risk was found between those treated in rural hospitals and those treated in urban nonteaching hospitals. Additionally, patients treated in the Midwestern (aOR = 0.83, 95% CI 0.73–0.95, *p* = 0.007) and Southern regions (aOR = 0.84, 95% CI 0.74–0.94, *p* = 0.002) had a lower mortality risk, whereas those treated in the Western region (aOR = 1.14, 95% CI 1.01–1.30, *p* = 0.040) had a higher mortality risk, when compared to those treated in the Northeastern region of the country. Diabetes Type 2 (aOR = 0.88, 95% CI 0.82–0.94, *p* < 0.001), nicotine use (aOR = 0.52, 95% CI 0.46–0.58, *p* < 0.001), and smoking (aOR = 0.58, 95% CI 0.52–0.63, *p* < 0.001) were associated with lower mortality risk. Conversely, atrial fibrillation (aOR = 1.69, 95% CI 1.58–1.82, *p* < 0.001) and heart failure (aOR = 1.61, 95% CI 1.50–1.73, *p* < 0.001) significantly increased mortality risk. Alcohol use and viral hepatitis were found not to be significant independent predictors of in-hospital mortality. These findings, detailed in Table 3, highlight the multifaceted nature of mortality risk in ischemic stroke discharges and underscore the importance of considering both clinical and socioeconomic factors in risk stratification and management strategies.

### 3.3. Liver Cirrhosis Leads to Worse Hospital Outcomes and Increased Resource Utilization in Ischemic Stroke Discharges

Discharges with concurrent liver cirrhosis and ischemic stroke demonstrated significantly different clinical courses and resource utilization patterns compared to those without cirrhosis. Cirrhotic discharges experienced longer hospital stays (6.91 vs. 5.47 days, aIRR = 1.35, 95% CI 0.65–2.05, *p* < 0.001) and incurred higher hospital charges ($98,421 vs. $82,086; aIRR = 13,074.69; 95% CI 4907.02–21,242.36; *p* = 0.002). They also exhibited a higher rate of hemorrhagic transformation (7.3% vs. 5.3%, aOR = 1.33, 95% CI 1.02–1.74, *p* = 0.038) and were less likely to be discharged home for self-care (30.7% vs. 36.0%, aOR = 1.35, 95% CI 1.15–1.57, *p* < 0.001).

Interestingly, no significant differences were observed between the groups in several critical care interventions and complications. These included mechanical ventilation requirements, vasopressor use, postthrombolysis hemorrhagic transformation, and mortality postthrombolysis. Similarly, discharge dispositions to home health care, short-term hospitals, skilled nursing facilities, or leaving against medical advice showed no significant differences.

These findings suggest that while liver cirrhosis is associated with increased resource utilization and certain complications in ischemic stroke discharges, it does not uniformly affect all aspects of care or outcomes. Table 2 provides a comprehensive overview of these outcomes for discharges with ischemic stroke, stratified by the presence or absence of liver cirrhosis.

## 4. Discussion

In this nationwide retrospective cohort study, we found that liver cirrhosis is a significant independent risk factor for increased mortality among discharges with ischemic stroke. Our findings demonstrate that ischemic stroke discharges with liver cirrhosis had a notably higher in-hospital mortality rate (7.1%) compared to those without cirrhosis (4.2%), with an aOR of 1.75 (95% CI: 1.32–2.32). This increased mortality risk aligns with previous studies, including a retrospective observational study in China that reported significantly higher in-hospital mortality in cirrhotic patients with ischemic stroke compared to those without ischemic stroke [11]. Similarly, Parikh et al. found a significant independent association between liver disease and adverse outcomes, including worse hospital discharge disposition and higher in-hospital mortality in stroke patients [12].

The impact of liver cirrhosis on stroke prognosis appears to be multifaceted. Cirrhosis is associated with endothelial dysfunction, systemic oxidative imbalance, increased inflammatory responses, and impaired hemostasis or hypercoagulability [6]. These factors may contribute to the increased risk of cardiovascular complications such as myocardial infarction, atrial fibrillation, and heart failure, which in turn may increase the risk of recurrent stroke or mortality [13].

Our study also revealed that cirrhotic discharges with ischemic stroke had longer hospital stays, higher hospital charges, and a higher rate of hemorrhagic transformation. These findings suggest that cirrhosis complicates the clinical course of ischemic stroke, potentially due to the complex interplay between hepatic dysfunction and cerebrovascular pathology. The coagulopathy associated with cirrhosis may contribute to the increased risk of hemorrhagic transformation, while the systemic effects of liver dysfunction may impair recovery and necessitate more intensive care.

### 4.1. Clinical Implications

Our findings have significant implications for the clinical management of ischemic stroke patients with comorbid liver cirrhosis. The increased mortality risk and higher incidence of complications in this population underscore the need for a tailored approach to their care. Clinicians should consider liver cirrhosis as a key factor in the prognostic assessment of ischemic stroke patients, prompting a more cautious outlook and potentially more intensive monitoring. Long-term follow-up may need to be more frequent, with aggressive secondary prevention strategies. These implications highlight the importance of recognizing liver cirrhosis as a significant comorbidity in ischemic stroke and adopting a more nuanced approach to care. There is a clear need for prospective studies to develop and validate specific management protocols for this high-risk group of patients.

### 4.2. Future Research Recommendations

Future research should focus on elucidating the pathophysiological mechanisms linking liver cirrhosis and ischemic stroke outcomes through prospective cohort studies and experimental designs. Clinical trials are needed to evaluate targeted interventions in cirrhosis management and their impact on stroke outcomes. Comparative effectiveness research should explore various treatment strategies, including reperfusion therapies and anticoagulation regimens, specifically for cirrhotic patients with stroke. Longitudinal epidemiological studies tracking trends in cirrhosis prevalence and its impact on stroke outcomes across diverse populations are essential for informing healthcare resource allocation. By addressing these research priorities, we can develop more effective, tailored strategies for managing ischemic stroke in patients with liver cirrhosis, ultimately improving outcomes for this high-risk group.

### 4.3. Strengths and Limitations of the Study

This study's strengths lie in its utilization of a large, nationally representative sample from the 2021 NIS database, providing comprehensive and current data. The application of multivariate regression analysis allows for adjustment of potential confounding factors, enhancing the robustness of our findings. The inclusion of various outcome measures offers a comprehensive understanding of liver cirrhosis's impact on ischemic stroke outcomes.

However, some limitations warrant consideration. The retrospective design precludes establishing causality, and reliance on administrative data may introduce coding errors and misclassification biases. The observational nature of the study limits our ability to control for all potential confounders, and the lack of detailed clinical information (e.g., severity of cirrhosis and stroke characteristics) may constrain the interpretation of results. Additionally, the study's cross-sectional nature prevents assessment of long-term outcomes or changes in risk over time.

Additionally, one notable finding in our study was the association between smoking and lower in-hospital mortality, a phenomenon referred to as the “smoker's paradox.” While previous research has documented this paradox in cardiovascular and stroke outcomes, its underlying mechanisms remain unclear. Potential explanations include differences in baseline characteristics or treatment strategies. Given the observational nature of our study, this association should not be interpreted as a causal protective effect, and further research is warranted to explore its implications.

Despite these limitations, our findings provide valuable insights into the relationship between liver cirrhosis and ischemic stroke outcomes, serving as a foundation for future prospective studies and targeted.

## 5. Conclusion

Our study provides robust evidence that liver cirrhosis is an independent and significant risk factor for increased mortality among patients hospitalized with ischemic stroke. These findings underscore the critical importance of recognizing and addressing liver cirrhosis in the management of ischemic stroke patients.

## Figures and Tables

**Table 1:** Characteristics of the study population.

**Variable**	**Cirrhosis cohort** **N** = 4464	**No cirrhosis cohort** **N** = 531735	**Total** **N** = 53619	**p** ** value**
Mean age (SD), in years	67.5 (10.8)	69.7 (13.7)	69.70 (13.7)	< 0.001
Gender				< 0.001
Female (%)	41.8	48.7	48.6	
Male (%)	58.2	51.3	51.4	
Hospital bed size				0.995
Small (%)	19.6	19.7	19.7	
Medium (%)	27.7	27.6	27.6	
Large (%)	52.7	52.7	52.7	
Race				< 0.001
Caucasian (%)	67.7	66.9	66.9	
Black/African American (%)	12.7	17.9	17.8	
Hispanic (%)	13.3	8.7	8.8	
Asian or Pacific Islander (%)	2.9	3.3	3.3	
Native American (%)	1.2	0.5	0.5	
Others (%)	2.2	2.7	2.7	
Mean Charlson comorbidity index (SD)	5.67 (2.3)	3.82 (2.1)	3.84 (2.1)	< 0.001
Insurance				< 0.001
Medicare (%)	63.1	62.9	62.9	
Medicaid (%)	16.2	10.4	10.4	
Private insurance (%)	14.5	19.4	19.3	
Self-pay (%)	3.0	4.2	4.2	
No charge (%)	0.2	0.3	0.3	
Other (%)	2.9	2.8	2.8	
Median household income for patient's ZIP code (socioeconomic status)				< 0.001
Quartile 1 (%)	33.3	31.0	31.0	
Quartile 2 (%)	26.7	25.5	25.5	
Quartile 3 (%)	22.8	23.6	23.6	
Quartile 4 (%)	17.2	19.9	19.9	
Comorbidities				
Hypertension (%)	44.7	55.5	55.4	0.001
Diabetes (%)	48.7	40.1	40.2	0.001
Type 1 diabetes (%)	0.8	0.6	0.6	0.594
Type 2 diabetes (%)	47.9	39.5	39.5	< 0.001
CKD (%)	26.5	19.4	19.5	0.001
Heart failure (%)	26.8	17.3	17.3	0.001
Smokers	18.4	19.6	19.6	0.370
Nicotine users	25.5	19.6	19.6	< 0.001

Abbreviations: CKD, chronic kidney disease; SD, standard deviation.

**Table 2:** Clinical outcomes of patients admitted with ischemic stroke with and without liver cirrhosis.

**Outcomes**	**Cirrhosis cohort** **N** = 4464	**No cirrhosis cohort** **N** = 531735	**aOR or IRR**	**95% confidence interval**	**p** ** value**
In-hospital mortality (%)	7.1	4.2	1.75	1.32–2.32	0.001
Mechanical ventilation (%)	4.9	3.5	1.20	0.86–1.66	0.283
Length of stay (mean)	6.91	5.47	1.35	0.65–2.05	0.001
Hospital charges (mean)	98,421.00	82,086.00	13,074.69	4907.02–21,242.36	0.002
Vasopressor requirement (%)	1.3	0.9	1.41	0.78–2.56	0.258
Hemorrhagic transformation (%)	7.3	5.3	1.33	1.02–1.74	0.038
Hemorrhagic transformation postthrombolysis (%)	3.1	8.2	0.42	0.10–1.73	0.229
Mortality postthrombolysis (%)	4.7	4.2	1.30	0.37–4.60	0.680
Discharge disposition					
a. Home					
-Self-care (%)	30.7	36.0	1.35	1.15–1.57	0.001
-Home health care (%)	18.3	18.2	1.05	0.88–1.26	0.563
b. Others					
-Short-term hospital (%)	2.7	2.4	1.08	0.72–1.63	0.698
-Skilled nursing facility, intermediate care facility, or other facility (%)	39.2	37.8	1.12	0.98–1.29	0.102
Against medical advice (%)	2.0	1.5	1.34	0.82–2.17	0.242

Abbreviations: aOR, adjusted odds ratio; IRR, incidence rate ratio.

**Table 3:** Predictors of in-patient mortality in patients admitted with ischemic stroke.

**Variable**	**aOR**	**95% lower CI**	**95% upper CI**	**p** ** value**
Liver cirrhosis	1.69	1.27	2.25	< 0.001
Female	0.91	0.85	0.97	0.005
Age				
18–44	Reference			
45–65	1.52	1.20	1.93	< 0.001
> 65	2.25	1.76	2.88	< 0.001
Median household income for patient's ZIP code (socioeconomic status)				
Quartile 1	Reference			
Quartile 2	0.89	0.82	0.98	0.015
Quartile 3	0.80	0.73	0.88	< 0.001
Quartile 4	0.64	0.57	0.71	< 0.001
Race				
Caucasian	Reference			
Black	0. 89	0.80	0.99	0.026
Hispanic	0. 81	0.71	0.92	0.002
Asian or Pacific Islander	0.99	0.84	1.18	0.948
Native American	1. 25	0. 69	2.24	0.457
Others	1.15	0.96	1.38	0.130
Insurance type				
Medicare	Reference			
Medicaid	1.22	1.06	1.40	0.005
Private insurance	1.07	0.96	1.20	0.220
Self-pay	0.80	0.36	1.82	0.600
Others	1.78	1.43	2.22	< 0.001
Diabetes				
Type 1 diabetes	1.14	0.76	1.72	0.525
Type 2 diabetes	0.88	0.82	0.94	< 0.001
Hospital location and teaching status				
Rural	Reference			
Urban nonteaching	0.95	0.80	0.95	0.007
Urban teaching	1.42	1.22	1.65	< 0.001
Hospital region				
Northeast	Reference			
Midwest	0.83	0.73	0.95	0.007
South	0.84	0.74	0.94	0.002
West	1.14	1.01	1.30	0.040
Nicotine use	0. 52	0.47	0.58	< 0.001
Smoking	0. 58	0.52	0.63	< 0.001
Atrial fibrillation	1.69	1.58	1.82	< 0.001
Heart failure	1.61	1.50	1.73	< 0.001
Alcohol use	0.92	0.77	1.11	0.393
Chronic viral hepatitis	1.23	0.75	2.03	0.410

Abbreviation: aOR, adjusted odds ratio.

## Data Availability

The data that support the findings of this study are available from the corresponding author upon reasonable request.

## Nomenclature

NIS: National Inpatient Sample
HCUP: Healthcare Cost Utilization Project
AHRQ: Agency for Healthcare Research and Quality
ORs: odds ratios
CI: confidence interval
STATA: statistical software used for data analysis
